# XRN2 Links Transcription Termination to DNA Damage and Replication Stress

**DOI:** 10.1371/journal.pgen.1006107

**Published:** 2016-07-20

**Authors:** Julio C. Morales, Patricia Richard, Praveen L. Patidar, Edward A. Motea, Tuyen T. Dang, James L. Manley, David A. Boothman

**Affiliations:** 1 Department of Neurosurgery, University of Oklahoma Health Science Center, Oklahoma City, Oklahoma, United States of America; 2 Department of Biological Sciences, Columbia University, New York, New York, United States of America; 3 Simmons Comprehensive Cancer Center, University of Texas Southwestern Medical Center, Dallas, Texas, United States of America; CABIMER, Universidad de Sevilla, SPAIN

## Abstract

XRN2 is a 5’-3’ exoribonuclease implicated in transcription termination. Here we demonstrate an unexpected role for XRN2 in the DNA damage response involving resolution of R-loop structures and prevention of DNA double-strand breaks (DSBs). We show that XRN2 undergoes DNA damage-inducible nuclear re-localization, co-localizing with 53BP1 and R loops, in a transcription and R-loop-dependent process. XRN2 loss leads to increased R loops, genomic instability, replication stress, DSBs and hypersensitivity of cells to various DNA damaging agents. We demonstrate that the DSBs that arise with XRN2 loss occur at transcriptional pause sites. XRN2-deficient cells also exhibited an R-loop- and transcription-dependent delay in DSB repair after ionizing radiation, suggesting a novel role for XRN2 in R-loop resolution, suppression of replication stress, and maintenance of genomic stability. Our study highlights the importance of regulating transcription-related activities as a critical component in maintaining genetic stability.

## Author Summary

Genomic instability is one of the primary causes of disease states, in particular cancer. One major cause of genomic instability is the formation of DNA double strand breaks (DSBs), which are one of the most dangerous types of DNA lesions the cell can encounter. If not repaired in a timely manner, one DSB can lead not only to cell death. If misrepaired, one DSB can lead to a hazardous chromosomal aberration, such as a translocation, that can eventually lead to cancer. The cell encounters and repairs DSBs that arise from naturally occurring cellular processes on a daily basis. A number of studies have demonstrated that aberrant structures that form during transcription under certain circumstances, in particular RNA:DNA hybrids (R loops), can lead to DSB formation and genomic instability, especially during DNA synthesis. Thus, it is important to understand how the cell responds and repairs transcription-mediated DNA damage in general and R loop-related DNA damage in particular. This paper both demonstrates that the XRN transcription termination factor links transcription and DNA damage, but also provides a better understanding of how the cell prevents transcription-related DNA damage.

## Introduction

Chromosomes are under constant assault by DNA damaging agents. These insults lead to a variety of DNA lesions [1] that include one of the most severe, the DNA double strand break (DSB) [2]. One DSB can be lethal, and if not repaired in a timely and accurate manner can lead to genomic instability and rearrangement, such as translocations, that can contribute to subsequent diseased states [2]. Genomic instability is recognized as one of the hallmarks of cancer [3]. It can arise from a variety of different mechanisms, eventually resulting in mutation or chromosomal aberrations leading to tumor formation or cell death [2]. One of the most common mechanisms leading to DSB formation and genomic instability is aberrant replication, which is found to be a major cause of disease, including cancer [4, 5]. The cell uses two major pathways, non-homologous end-joining (NHEJ) and homologous recombination (HR), to repair DSBs [2].

A number of studies over the last decade have provided evidence that a major source of genomic instability and DSB formation during replication is mediated by transcription, and links between transcription and genomic instability are becoming more apparent [6–9]. In some cases, genomic instability is caused by collisions between the replication and transcriptional machineries, and resultant RNA:DNA hybrids, or R loops [10]. R loops are a consequence of transcription that can form under a variety of conditions and if not properly resolved lead to DSBs and genomic instability [7, 9]. However, transient R-loop formation is an essential step during certain cellular processes such as immunoglobulin class switch recombination and in some cases RNA polymerase II (RNAPII) transcription regulation and termination [11–14].

Transcription termination by RNAPII is an incompletely understood process that requires multiple protein factors [15]. Foremost amongst these are components of the cleavage/polyadenylation machinery, consistent with the long-known requirement of an active polyadenylation signal for subsequent termination [15, 16]. Other factors involved in termination include: (i) XRN2, a 5’-3’ exoribonuclease that performs a key function in termination by degrading nascent RNA downstream from the 3’ cleavage site [12, 17, 18]; recent studies have provided evidence that XRN2 functions in termination of most RNAPII transcripts [19] (ii) PSF, which together with p54(nrb) works to recruit XRN2 to pre-determined sites within the genome [20]; (iii) Kub5-Hera (K-H), which facilitates localization of XRN2 along the genome [21]; and (iv) Senataxin (SETX), an RNA:DNA helicase that in some cases is required to unwind the nascent RNA from its DNA template to allow for its degradation by XRN2 [12].

Interestingly, along with roles in transcription termination, several of the above factors have been implicated in the DNA damage response (DDR) and DSB repair. PSF and p54(nrb) have functional roles in both HR and NHEJ [22, 23]. Loss of PSF or p54(nrb) leads to increased DSB formation, abrogated ATM signaling, delayed DSB repair kinetics and hypersensitivity to ionizing radiation (IR) [24, 25]. Cells deficient in K-H expression display increased R-loop and DSB formation, abrogated NHEJ DSB repair via reduced expression of the DNA endonuclease Artemis, delayed DSB repair kinetics, hypersensitivities to IR and other DSB-inducing agents, and genomic instability [21]. SETX is involved in resolving R loops that form during transcription and lead to DSBs [26–29]. To date, however, a role for XRN2 in the DDR has not been suggested.

In this study, we employed genetic, biochemical and cell biological techniques to uncover novel functions of XRN2 in R-loop resolution, DNA damage signaling and repair. Indeed, we found that XRN2 undergoes nuclear re-localization in response to genomic insults, particularly after exposure to ultraviolet (UV) and γ-irradiation (IR). Importantly, we found that relocation of XRN2 is dependent on active transcription and R-loop formation. Cells lacking XRN2 demonstrate increased levels of R loops, DSBs, particularly at transcriptional pause sites, genomic instability and are hypersensitive to DNA damaging agents. Loss of XRN2 adversely affects the NHEJ pathway of DNA repair. Finally, XRN2-deficient cells demonstrate increased levels of replication stress and an abrogated DNA repair capability after genomic insult.

## Results

### XRN2 undergoes DNA damage-inducible nuclear re-localization

**Fig 1:**
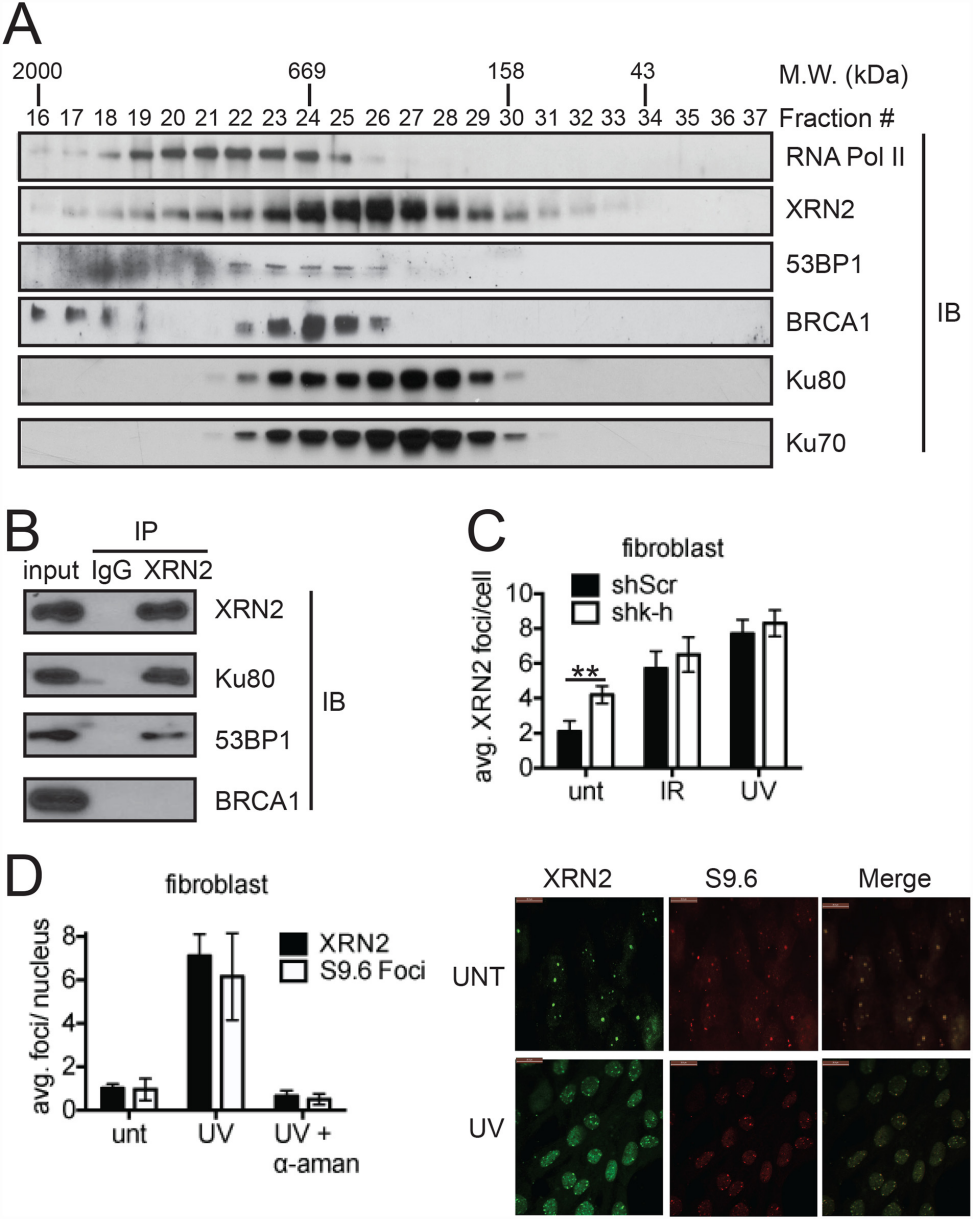
XRN2 is a DNA damage-responsive factor. (**A**) To interrogate potential *in vivo* XRN2-interacting partners, exponentially growing HeLa cells were collected and lysed for FPLC analyses. Individual fractions were separated by SDS-PAGE and indicated proteins visualized by immunoblot (IB). (**B**) Interactions between XRN2 and the DNA damage regulators Ku80, 53BP1, and BRCA1 were interrogated by immunoprecipitation (IP). (**C**) XRN2 foci were quantitated in human fibroblasts stably expressing an shRNA control (shScr) or a Kub5-Hera shRNA (shk-h) in mock (unt)-, IR (1 Gy)-, and UV (20 J/m^2^)-treated cells. (**D**) Sub-cellular XRN2 localization and S9.6 foci were visualized in mock-, UV (20 J/m^2^)-, or UV (20 J/m^2^)- + α amanitin (α-aman)-treated shScr fibroblast cells by immunofluorescence. (*p<0.05).

We first investigated whether XRN2 associated with known DNA repair factors. To this end, we performed gel filtration chromatography using HeLa whole-cell extracts. The elution pattern of XRN2 (fractions 23–29) coincided with the patterns of the DNA damage repair proteins 53BP1, Ku70/80 and BRCA1 (Fig 1A). Similar to XRN2, several DNA repair factors have been found to interact with SETX [27–31]. Among them, BRCA1 mediates SETX recruitment to a subset of transcription pause sites, and aids in SETX-mediated DNA repair [29]. To support the idea that XRN2 interacts with DNA repair proteins, we performed immunoprecipitation using XRN2-specific antibodies. Indeed, we found that 53BP1 and Ku80 both immunoprecipitated with XRN2 (Fig 1B). Unlike what was observed with SETX, we did not detect BRCA1 after XRN2 immunoprecipitation. Notably, Ku80 has also been found to interact with SETX in an affinity purification of FLAG-tagged SETX [31]. These data suggest a possible role for XRN2 in responding to DNA damage, particularly in the NHEJ pathway.

Several DNA damage regulators, such as 53BP1 and γ-H2AX, form discrete foci after genomic insults [32–34]. We next examined whether XRN2 formed DNA damage-induced foci. Indeed, XRN2 displayed foci formation in response to both IR and UV exposures (Fig 1C and S1A, S1B and S1C Fig). We observed an average of ~2 XRN2 foci in untreated cells compared to ~6–8 XRN2 foci in IR- or UV-treated cells (Fig 1C). Coincidently, a recent proteomics analysis demonstrated that XRN2 undergoes DNA damage-inducible phosphorylation in response to UV and IR treatments [35]. Importantly, along with purifying 53BP1 by immunoprecipitation, XRN2 foci co-localized with 53BP1 after genomic insult (S2 Fig), further supporting the idea that these two proteins associate with one another. Using previously described human fibroblasts infected with a Kub5-Hera specific shRNA (shk-h) [21], we found that XRN2 foci formation in response to DNA damage was independent of K-H expression (Fig 1C and S3 Fig). This contrasts with XRN2 localization to the 3’ end of genes, where K-H is required [21].

It was recently demonstrated that UV damage leads to the formation of R loops [36]. Interestingly using the S9.6 antibody, which recognizes RNA:DNA hybrids [37], in conjunction with XRN2 antibodies, we observed that foci formation for both RNA:DNA hybrids and XRN2 were significantly increased after UV exposure (Fig 1D). We also observed that XRN2 formed foci after UV damage with kinetics closely mirroring R-loop formation, while there was no change in DSB foci, marked by 53BP1 and γ-H2AX staining (S4 Fig). These observations, and the fact that XRN2 foci also co-localized with R-loop foci (Fig 1D), suggest that XRN2 is recruited to R loops or stalled RNAPII rather than to DSBs. When cells were treated with the RNAPII inhibitor α-amanitin both XRN2 and R-loop foci failed to form after UV treatment (Fig 1D). These data strongly suggest that R-loop formation and active transcription are both required for XRN2 foci formation after genomic insult.

### Loss of XRN2 results in increased DSBs and genomic instability

**Fig 2:**
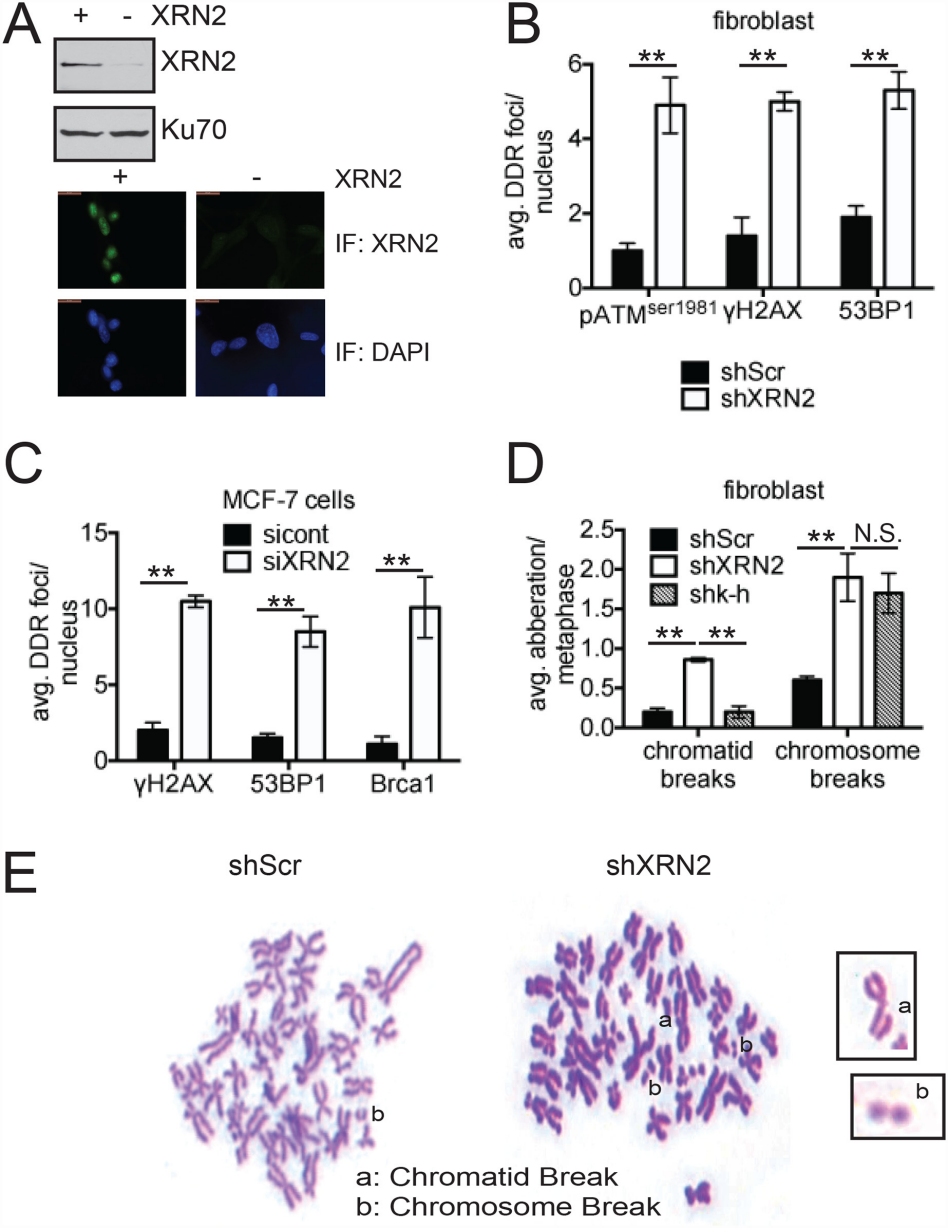
Loss of XRN2 leads to increased DSB formation and genomic instability. (**A**) Steady state levels of XRN2 protein in shScr (+) compared to shXRN2 (-) fibroblast cells were monitored by western blotting and immunofluorescence. (**B, C**) Basal levels of 53BP1, pATM^ser1981^, γ-H2AX, and BRCA1 foci/nuclei were quantitated in shScr, shXRN2, and MCF-7 cells treated with control and XRN2 specific siRNA. (**D, E**) Genomic aberrations were quantified in shScr, shXRN2, and shk-h cells using derived metaphase spreads. (**p<0.01).

We next wished to examine a potential role for XRN2 in the DDR. For this, we employed an XRN2-specific shRNA to generate an immortalized human fibroblast cell line with lowered XRN2 expression levels (shXRN2), and a non-targeting scrambled sequence shRNA to generate comparable control cells (shScr). We also reproduced our results using XRN2 siRNA in MCF-7, an ER+PR+ breast cancer cell line [38]. We verified the decrease in steady-state levels of XRN2 protein in shXRN2 cells compared to shScr cells by both western blot and immunofluorescence (IF) (Fig 2A). We previously showed that loss of K-H and p54(nrb), two factors implicated in mediating XRN2 genomic distribution, led to increased DSB formation [21]. Similar to K-H- and p54(nrb)-deficient cells, we observed an increased level of 53BP1, γ-H2AX, ATM pSer 1981, and BRCA1 foci/nuclei in XRN2 siRNA-treated MCF-7 cells and in the XRN2 shRNA-expressing fibroblasts (Fig 2B, 2C and S5A–S5D Fig). We also found an increase in the amount of Rad51 foci (S6A Fig) in shXRN2 cells compared to controls, suggesting that cells depleted of XRN2 are subjected to an increased level of basal DNA damage.

We next examined the ability of shScr and shXRN2 fibroblasts to perform NHEJ. We used for this a previously published plasmid-based NHEJ assay [39]. This assay employed a linearized GFP reporter plasmid, generated by HindIII digestion, leading to a compatible DNA end or I-SceI digestion, resulting in incompatible DNA ends due to restriction site orientation. Significantly, compared to shScr cells, shXRN2 cells could not efficiently repair either compatible or incompatible DNA ends, indicating that loss of XRN2 abrogated the ability of cells to repair DSBs via the NHEJ pathway (S6B Fig). Previously, we showed that K-H-deficient cells (shk-h) also lacked the ability to perform NHEJ, but only at non-compatible DSB ends, through loss of Artemis expression [21]. Comparative western blot analyses in shScr, shXRN2 and shk-h cells revealed that loss of XRN2 did not result in a concurrent Artemis loss (S6C Fig), illustrating a significant difference between the two transcription termination factors.

We next performed metaphase spreads to examine cytogenetically the extent of genomic instability in the shXRN2 fibroblasts compared to shScr cells. Consistent with increased DSBs and apparent loss of DSB repair ability of XRN2-deficient cells, we noted that shXRN2 cells harbored increased amounts of both chromatid and chromosome type breaks versus shScr cells (Fig 2D and 2E). When we compared shXRN2 with shk-h cells we found similar levels of chromosome-type damage, but loss of XRN2 led to significantly more chromatid-type damage, which was not seen in K-H deficient cells (Fig 2D), again suggesting an important difference between loss of XRN2 and K-H.

### Loss of XRN2 sensitizes cells to a wide variety of genomic insult

**Fig 3:**
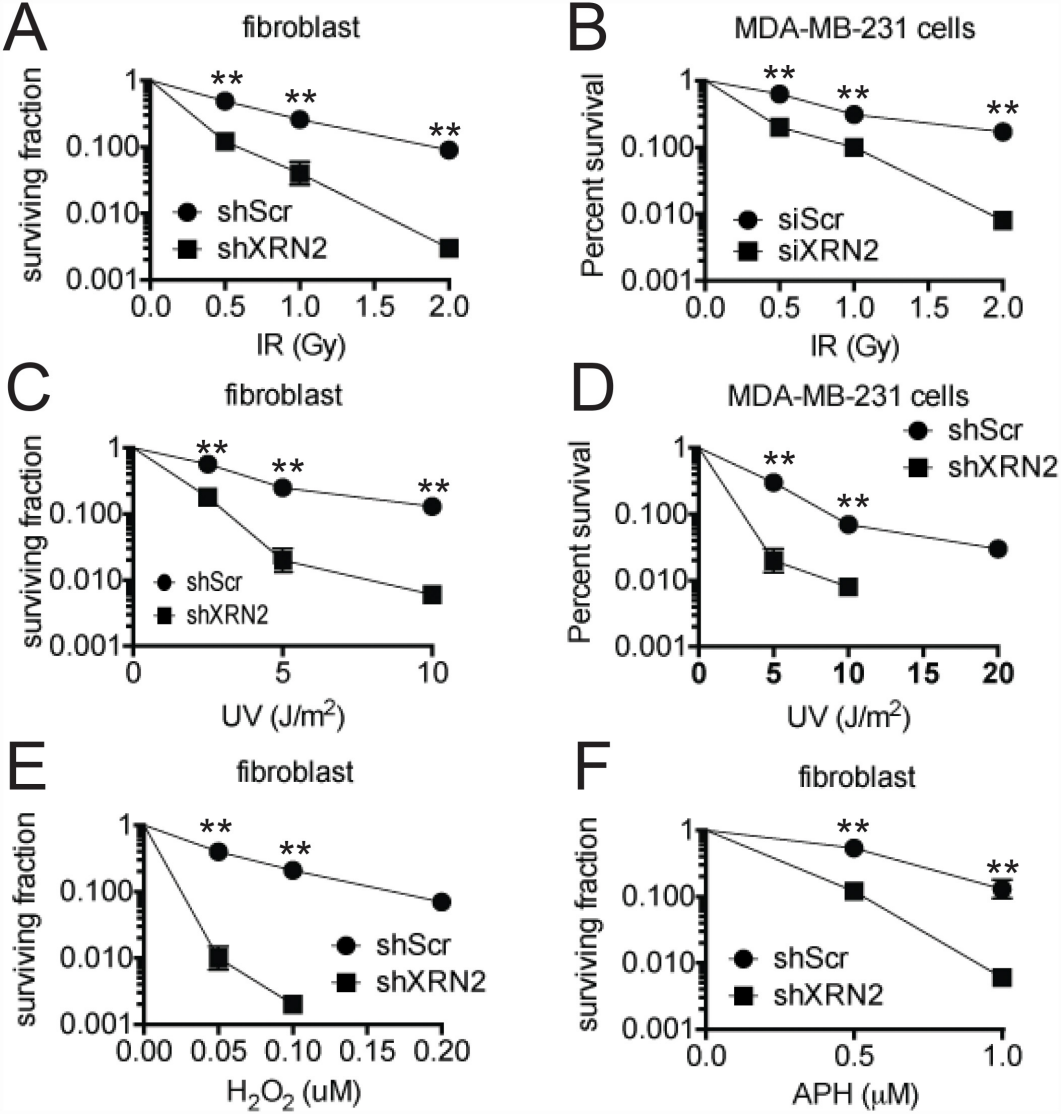
XRN2-deficient cells are hypersensitive to various chemotherapeutic agents. (**A-D**) shScr and shXRN2 fibroblasts and MDA-MB-231 cells transfected with a siRNA control or targeting XRN2 were either mock-treated or exposed to: (**A, B**) ionizing radiation (IR); (**C, D**) ultraviolet light (UV). (**E-F**) shScr and shXRN2 fibroblasts were either mock-treated or exposed to: (**E**) H_2_O_2_ or (**F**) Aphidicolin (APH). Cells were then monitored for survival using colony forming assays. Colonies of >50 normal-appearing cells were quantified for mock- versus agent-exposed cells. (**p<0.01).

Cells deficient in XRN2 displayed increased DSBs and genomic instability along with decreased DNA repair capacity. Interestingly, this is similar to previously published reports on K-H and PSF, two factors important in mediating XRN2 distribution along the genome [20, 21, 24]. Cells deficient in K-H or PSF expression also demonstrated hypersensitivities to DNA damaging agents, such as IR [21, 24]. Similarly, XRN2 deficient cells, fibroblast or MDA-MB-231 cells, a triple negative breast cancer cell line [38], were hypersensitive to various genomic insults as illustrated by decreased colony forming ability after exposure to IR, aphidicolin (APH) or hydrogen peroxide (H_2_O_2_) (Fig 3A, 3B, 3E and 3F). Notably, both MDA-MB-231 and fibroblast XRN2-depleted cells were also hypersensitive to UV radiation (Fig 3C and 3D). These results reveal a difference between XRN2- and SETX-deficient cells, which show sensitivity to oxidative DNA damage but not IR, and also confirm a difference between XRN2 and K-H deficient cells, which are sensitive to IR, but not UV [21, 40].

### Cells lacking XRN2 undergo increased replication stress

**Fig 4:**
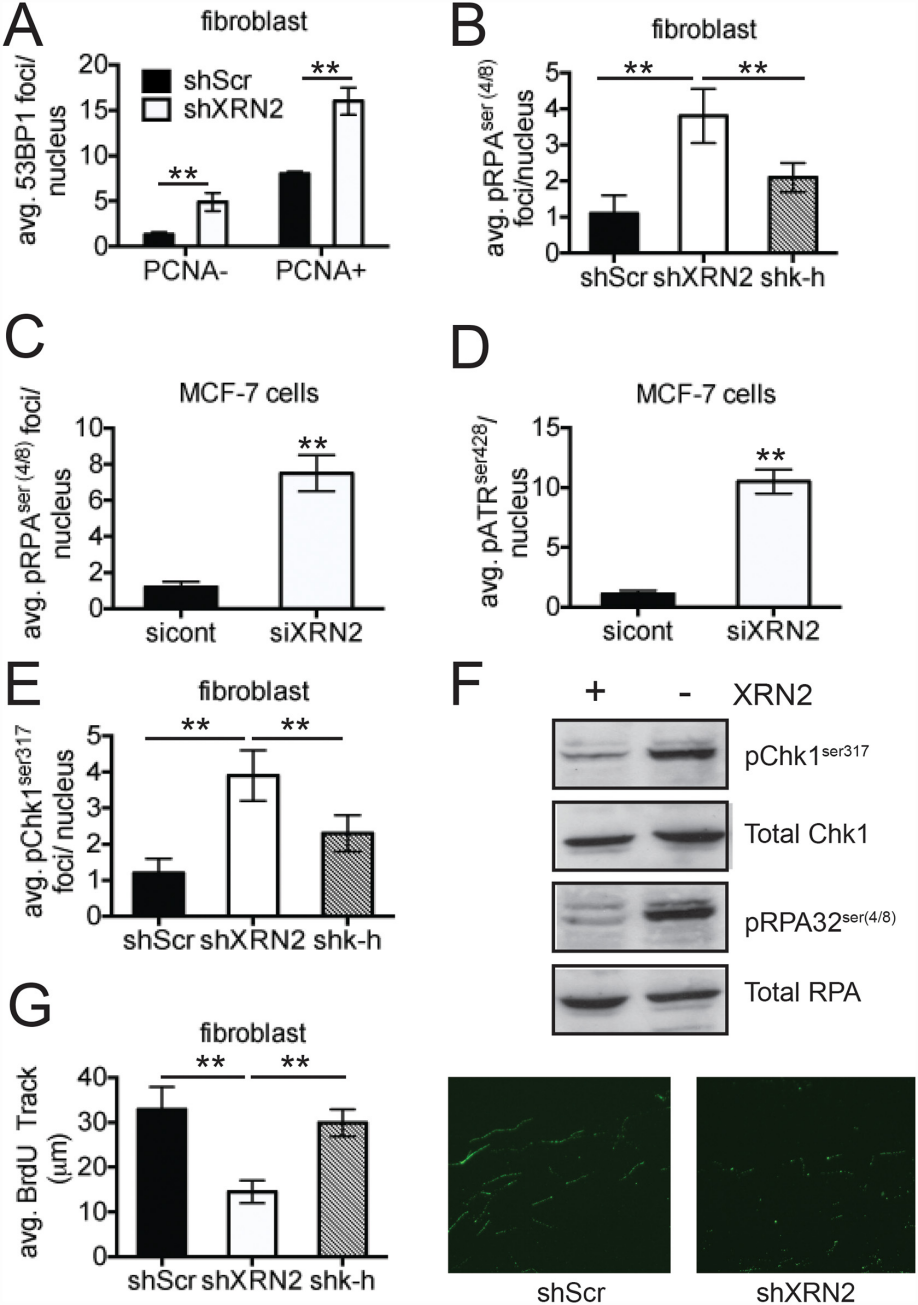
Loss of XRN2 leads to increase amounts of replication stress. (**A**) Basal levels of 53BP1 were monitored in PCNA positive cells, an S-phase indicator, in shXRN2 and shScr cells by immunofluorescence (IF). (**B, C**) Basal levels of phosphorylated RPA, were monitored by IF in shXRN2, shScr, shk-h fibroblasts and MCF-7 cells transfected with control or XRN2 specific siRNAs. (**D**) Basal levels of phosphorylated ATR were monitored by IF in MCF-7 cells transfected with a siRNA control and a siXRN2. (**E**) Basal levels of phosphorylated Chk1 were monitored in.shScr, shXRN2, and shk-h fibroblasts by IF. (**F)** Basal levels of phospho-Chk1-pS-317 and RPA32-pS-(4/8), replication stress indicators, in shXRN2 (-) versus shScr (+) cells were monitored by western blotting. (**G**) DNA replication elongation was monitored in log-phase shScr, shXRN2, or shk-h fibroblasts by DNA fiber analyses. (**p<0.01).

As shown above, cells lacking XRN2 display increased amounts of chromatid damage. This observation suggested that loss of XRN2 may adversely affect cells during DNA replication, as chromatid-type aberrations originate due to DNA damage occurring during S and G_2_ phases of the cell cycle [41]. Loss of XRN2 also leads to the focal accumulation of several factors required for homologous recombination, such as ATM, BRCA1 and Rad51 (Fig 2B, 2C and S6A Fig). These results suggest that loss of XRN2 can cause replicative stress. Initial experiments revealed that the shXRN2 fibroblasts displayed increased 53BP1 foci formation compared to shScr cells (Fig 4A) in cells expressing PCNA, a marker of cells undergoing DNA synthesis. We also observed increased phosphorylation of RPA32, activated ATR, and the checkpoint kinase CHK1 in both fibroblast and MCF-7 cells (Fig 4B–4F and S7A, S7B Fig), all indicators of replication stress. To measure replication fork impairment in shXRN2 cells directly, we performed DNA fiber analyses and found that nucleotide (BrdU) incorporation in shXRN2 cells was ~50% less than in shScr cells (15 μm vs 30 μm, respectively) (Fig 4G). Altogether, these data demonstrate that XRN2-deficient cells undergo significantly increased replication stress.

### Loss of XRN2 results in increased R-loop formation

**Fig 5:**
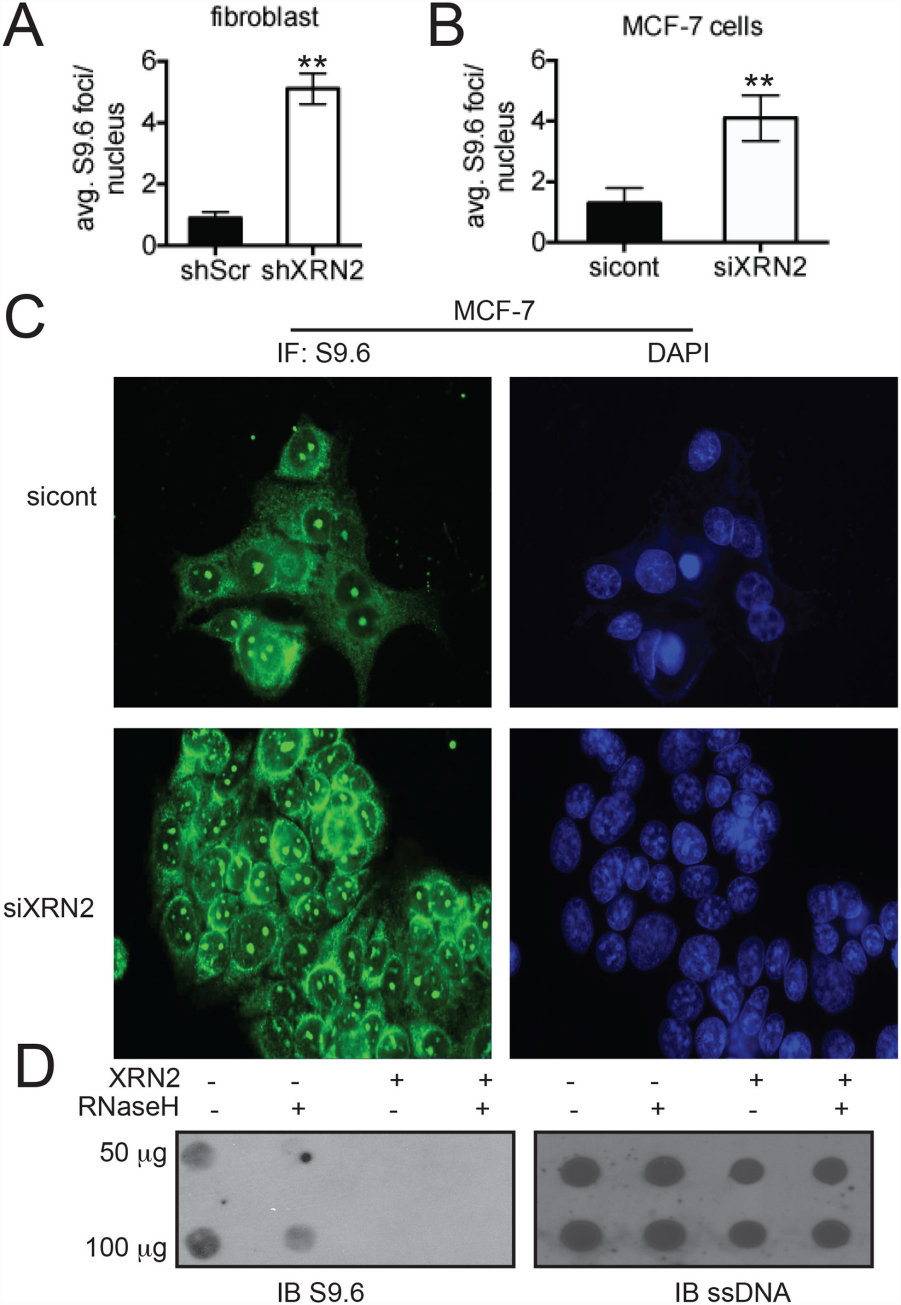
XRN2-deficient cells display-increased amounts of R loops. (**A-C**) Basal levels of R loops were monitored and quantitated by immunofluorescence with S9.6 antibody in shXRN2 and shScr fibroblasts and MCF-7 cells transfected with a siRNA control (sicont) or targeting XRN2 (siXRN2). (**D**) Levels of nuclear R-loop formation in control and XRN2 KD cells as indicated examined by dot-blot analysis using the S9.6 antibody. (**p<0.01).

A possible explanation for the observed increase in DNA damage and replication stress is that depletion of XRN2 leads to excess R-loop formation. To investigate this, we examined XRN2 knocked-down (KD) cells for basal levels of R-loop formation by IF, using the S9.6 antibody. Indeed, MCF-7 and fibroblast cells deficient in XRN2 exhibited an ~4-fold increase in R loops versus control cells (Fig 5A–5C). One caveat to measuring R loops by IF is that it may be difficult to distinguish between R loops formed in nuclear DNA to those formed in other sub-nuclear compartments, such as the nucleolus [7, 42]. However, it has been observed that R loops that form in the nucleus tend to be sensitive to RNaseH, while R loops within the nucleolus tend to be RNaseH resistant [43]. To support the notion that loss of XRN2 leads to increased R-loop formation within nuclear DNA, we isolated genomic DNA from MCF-7 cells with and without XRN2 and performed dot blot analysis. Again, we found an increase in the amount of S9.6 signal with the genomic DNA of MCF-7 cells depleted of XRN2 as compared to control cells (Fig 5D). Importantly, the S9.6 signal was strongly diminished after RNaseH treatment. All samples used in the dot blot analysis were treated with RNaseA, to remove any free RNA that the S9.6 antibody may cross react with [44] and an antibody against single-stranded DNA was used to ensure equal loading of each sample after DNA denaturation (Fig 5D).

To determine how transcription and R-loop formation contribute to the DNA damage observed in shXRN2 cells, we treated shScr and shXRN2 cells with α-amanitin or transfected each cell line with GFP control or GFP-RNaseH expression plasmids to remove R loops, and then measured the number of 53BP1 foci. While we observed the expected increase in 53BP1 foci in mock-treated or GFP-transfected shXRN2 cells, we found that either inhibition of transcription (with α-amanitin) or removal of R loops (with RNaseH-GFP expression) led to decreased 53BP1 foci in shXRN2 cells, to levels comparable to shScr cells (S8A and S8B Fig). The decrease in 53BP1 foci in shXRN2 cells after α-amanitin treatment correlated well with the decrease in R-loop levels visualized in these cells (S8C Fig). These data confirm a role for XRN2 in R-loop removal and protection from DSB accumulation.

### R-loop formation inhibits DNA repair after genomic insult

**Fig 6:**
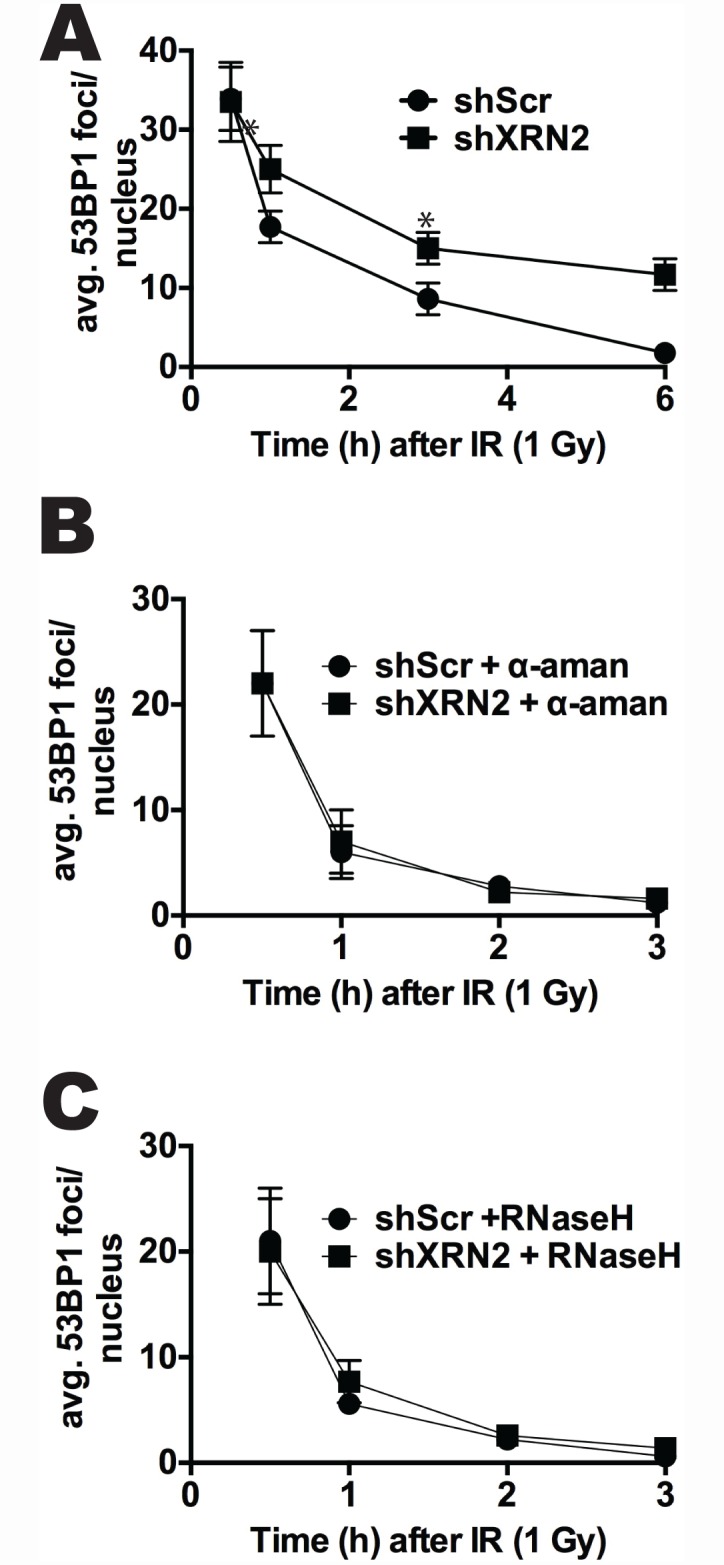
R-loop formation and transcription contribute to delayed DSB repair kinetics in XRN2-deficient cells. **(A)** Levels of R loops were monitored in mock- or IR (1 Gy)-exposed shXRN2 compared to shScr fibroblast cells by immunofluorescence. **(B-C)** Regression of 53BP1 foci/nucleus was monitored by IF in IR (1 Gy)-treated shXRN2 and shScr cells that were exposed to α-amanitin (α-aman) or transfected with GFP-RNaseH. (*p<0.05).

Since the loss of XRN2 sensitized cells to IR treatment (Fig 3A), we examined the effects of IR on R-loop formation in shXRN2 cells. Because R loops can directly lead to DSBs, we examined whether active transcription or R-loop formation directly affected DNA repair (regression of 53BP1 foci) after IR treatment. shScr or shXRN2 cells were treated or not with α-amanitin or transfected with GFP- or GFP-RNaseH expression plasmids prior to IR exposure and 53BP1 foci/nuclei regression kinetics were assessed at various times after IR exposure. Interestingly, a distinct and significant delay in the disappearance of IR-treated-induced 53BP1 foci in shXRN2 compared to shScr cells was observed, suggesting a defect in DNA repair kinetics (Fig 6A). However, inhibition of transcription by α-amanitin or removal of R loops by RNaseH completely restored DNA repair kinetics after IR exposure in shXRN2 cells (Fig 6B and 6C), suggesting that XRN2-deficient cells, along with an inability to properly perform DSB repair through the NHEJ pathway, are defective in R-loop resolution after IR.

### Loss of XRN2 leads to accumulation of DDR proteins at 3’ transcriptional pause sites of genes

**Fig 7:**
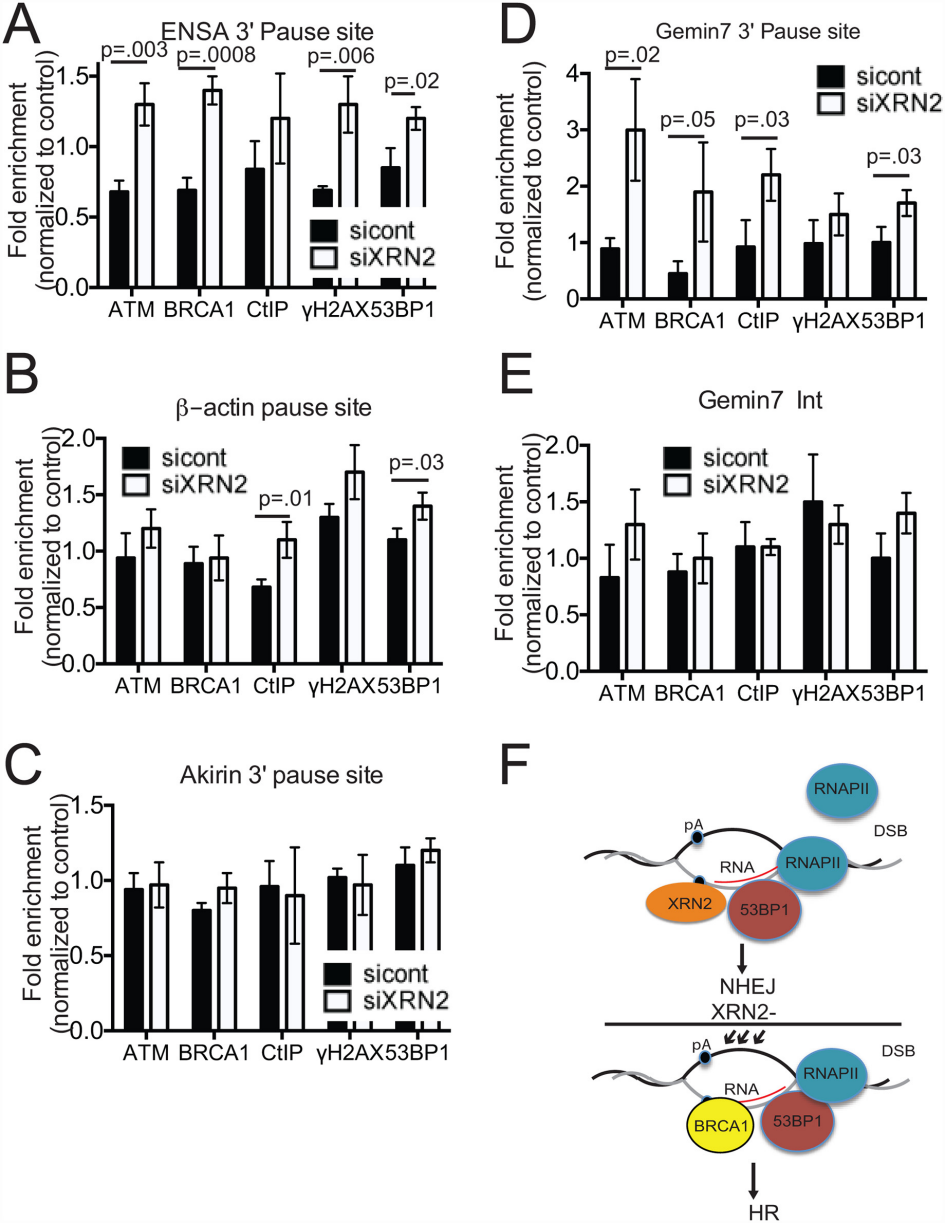
DDR regulators accumulate at the 3’ end of genes after XRN2 loss. (**A-E**) Accumulation of the DNA damage regulators, ATM, BRCA1, γ-H2AX, 53BP1, and CtIP, was monitored at the 3’ pause sites of the (**A**) *ENSA*, (**B**) *β-actin*, (**C**) *Akirin*,*1* (**D**) *Gemin7* genes and (**E**) an intronic region of *Gemin7* by chromatin immunoprecipitation. n = 3, S.E. is indicated. (**F**) Model for XRN2 functions in DNA repair pathway choice. In normal conditions, XRN2 binds to the NHEJ factor 53BP1 promoting DSB repair via the NHEJ pathway. In the absence of XRN2, NHEJ is inhibited downstream of 53BP1, allowing DSB repair via the HR pathway.

Lastly, we examined whether DDR factors accumulate at transcriptional pause sites on genes that undergo R-loop-dependent termination in XRN2-depleted cells. To do this, we performed chromatin immunoprecipitation (ChIP) to assay the presence of several DDR proteins at the 3’-ends of three genes subject to R-loop-dependent termination, *ENSA*, *Gemin7* and *β-actin*, and also *Akirin 1*, which are R-loop-independent [12, 29, 45]. Strikingly, we found that XRN2 depletion in HeLa cells led to accumulation of ATM, BRCA1, CtIP, 53BP1 and γ-H2AX at the termination pause site of the *ENSA* gene (Fig 7A), to an enrichment of ATM, BRCA1, CtIP, and 53BP1 at the *Gemin7* pause site (Fig 7D) and to a lesser extent, to accumulation of CtIP and 53BP1 at the 3’-end of the *β-actin* gene (Fig 7B). We detected no enrichment of any of the DDR factors at the 3’ end of the *Akirin 1* gene after XRN2 KD (Fig 7C). We also examined accumulation of the same DDR factors at an intronic region of *Gemin7* and did not detect significant changes after XRN2 loss (Fig 7E). These data suggest that XRN2 plays an important role in maintaining genomic integrity at the 3’ pause sites of genes. We note that SETX, which as mentioned above can function in termination by resolving R loops located downstream of certain poly(A) signals, has also been implicated in the DDR [26–28, 40]. In some cases through an interaction with BRCA1 at specific transcriptional pause sites including the three analyzed above [29]. Indeed, similar to the loss of XRN2, we observed that SETX KD led to slightly increased 53BP1 foci (S9A Fig) and initiation of ATM-mediated DNA damage signaling, as measured by increases in Chk2 and H2AX phosphorylation (S9B Fig). In light of these findings, we wished to eliminate the possibility that a concurrent loss of SETX following XRN2 depletion might underlie the effects we have attributed to XRN2. We measured SETX levels after XRN2 KD by Western blot and found that SETX expression was not altered (S9C Fig). Additionally, SETX immunoprecipitation failed to co-purify XRN2, while the known SETX-interacting partner Rrp45 [26] was detected (S9D Fig). Together, our data indicate that XRN2 plays an important role in protecting cells from DNA damage accumulation at termination pause sites of a subset of genes.

## Discussion

Regulation of transcription is a critical process essential for cell survival. A consequence of active transcription is the occasional formation of R loops [7]. While R loops can naturally form during transcription, their overly prolonged existence or aberrant formation can be a severe threat to genomic integrity [7]. Therefore, elucidating mechanisms by which cells prevent inappropriate R-loop formation and insure their resolution is imperative to understanding how genomic stability is maintained. Our results provide novel insights into how this occurs, unexpectedly implicating the 5’-3’ exoribonuclease XRN2 in this process. XRN2 functions in RNAPII transcription termination by degrading nascent RNA downstream of the poly(A) cleavage site [17]. Our data suggest a requirement of XRN2 in preventing formation of R loops, likely functioning at terminator regions downstream of 3’ cleavage sites, where R loops have indeed been detected [12, 14, 29]. Thus a conclusion from our data is that XRN2 is required to ensure that these R loops do not persist or perhaps simply reform after being resolved by SETX. Below we discuss both how XRN2 exerts this function, as well as the newly discovered role for XRN2 in the DDR.

Our data has implicated XRN2 as a significant factor in helping cells prevent DNA damage and maintain genomic stability. For example, we showed that loss of XRN2 leads to increased hypersensitivity to ionizing radiation. This is a characteristic seen in cells that have lost factors involved in DNA repair, especially those involved in NHEJ [46, 47]. Consistent with this, we observed an interaction between XRN2 and factors involved in the NHEJ pathway of DSB repair, such as 53BP1 and Ku80. Additionally we found that loss of XRN2 leads to accumulation of factors involved in the HR pathway of DSB repair, such as BRCA1 and CtIP, to the 3’ transcriptional pause site of genes undergoing R-loop-dependent transcription termination [12, 29]. It is worth noting that we detected BRCA1 at 3’ pause sites only after XRN2 KD, while Hatchi et al. found BRCA1 at the same pause sites in normal cells [29]. Although the basis for this difference remains to be determined, our data support the conclusion that this tumor suppressor, XRN2, functions in the DDR at regions of R-loop-associated transcription termination.

Our findings suggest that XRN2 plays a role in DNA repair pathway choice at sites of R-loop-induced DNA damage. Accumulation of CtIP at termination pause sites we observed after XRN2 KD suggests that DNA end-resection has occurred and that the HR repair pathway has been initiated [48]. However, accumulation of 53BP1 at the same sites is intriguing because it is believed that 53BP1 and BRCA1 are antagonistic to one another, with 53BP1 promoting NHEJ repair, and BRCA1 and CtIP promoting HR repair [49, 50]. Thus we suggest that XRN2 favors the use of NHEJ repair factors through its interactions with 53BP1, Ku70 and Ku80, while loss of XRN2 leads to the recruitment of factors involved in the HR DSB repair pathway (see model, Fig 7F). This model however does not explain why 53BP1 still accumulates at pause sites after XRN2 KD. Since 53BP1 foci increase after XRN2 KD, an interesting possibility is that 53BP1 recruits or stabilizes XRN2 to these sites in a similar way that BRCA1 recruits SETX (29). Indeed, Hatchi et al. showed that while BRCA1 KD impaired SETX recruitment to 3’ pause sites, SETX KD did not affect BRCA1 accumulation. Since XRN2 interacts with 53BP1 and Ku 80, but not BRCA1, and loss of XRN2 decreases the cell's ability to repair DSBs via NHEJ, we propose that XRN2 acts as a scaffolding protein, facilitating recruitment of factors downstream of 53BP1, such as Ku70 and Ku80, to the DSB site, thus promoting NHEJ repair. We cannot, however, exclude the possibility that XRN2 interaction with NHEJ factors is an R-loop-independent process that plays a role in global NHEJ, as suggested by the results of our GFP plasmids assays.

In addition to its function at gene 3’ ends, SETX has also been implicated in detecting and regulating R loops occurring after replication stress. Yüce and West demonstrated that SETX forms discrete nuclear foci and co-localizes with 53BP1 and γ-H2AX after aphidicolin treatment [28]. SETX has also been shown to interact with factors required for both HR and NHEJ, such as BRCA1, DNA-PKcs, Ku70 and Ku80 as well as Mre11 [28–31]. Furthermore, Richard *et al*. provided evidence that SETX, in a sumoylation-dependent manner, interacts with the exosome complex and recruits it to sites of transcription-replication collisions [26]. The exosome is a multisubunit complex containing a 3’ to 5’ exoribonuclease activity and is involved in mRNA turnover and RNA quality control [51]. Importantly, previous studies in human and yeast have also suggested that the exosome can play a role in the DDR and prevention of genome instability [26, 52, 53].

The above observations, together with the data presented here, suggest two related mechanisms by which cells resolve R loops and thereby prevent R loop-mediated DNA damage. One is that SETX and the exosome cooperate to disrupt R loops formed during transcriptional elongation and/or replication stress [26, 30]. In this scenario, SETX would resolve the RNA/DNA hybrid that forms behind a stalled RNAP II and the exosome then degrades the RNA from the 3’ end released from the transcription bubble. This would prevent the RNA from possibly reforming the R loop or causing other deleterious effects. In another mechanism, we propose that SETX and XRN2 function in the resolution of R loops at or near certain transcription termination sites. Following endonucleolytic cleavage of the pre-mRNA at the polyA site, the downstream RNA containing a 5’ monophosphate is degraded by XRN2 as part of the termination process for most RNAPII transcripts [17, 20]. In some situations, depending on the susceptibility of the sequence to R-loop formation, SETX is also required for termination, to resolve R loop structures that may block XRN2 [17]. In these instances, SETX and XRN2 work together to degrade the RNA at sites of R-loop formation. In the absence of XRN2, not only would termination be blocked but the R loop could also reform, leading to DNA damage we have described. It is not unlikely that both mechanisms co-exist at some sites of R-loop formation, leading to a 5’-3’ (through XRN2) and a 3’-5’ (through the exosome) degradation of the RNA moiety.

In summary, our results have shown that XRN2, previously known to function in transcription termination and RNA turnover, also has an important role in the DNA damage response. Thus, our findings have provided further evidence for the importance of controlling RNA metabolism for maintenance of genomic stability.

## Materials and Methods

### Cell culture

shScr, shXRN2, and shk-h cells derived from immortalized human fibroblasts, were generated using lentiviral shRNA constructs as described [21] under occasional selection with 1 μg/ml puromycin. They were grown in DMEM with 15% FBS, L-glutamine, 100 μg/ml hygromycin, and 1 μg/ml puromycin in a 10% CO_2_-90% O_2_ humidified air atmosphere at 37°C. HeLa cells were also used to derive a matched set of shScr and shSETX cells.

### Antibodies used

Antibody recognizing 53BP1 (A300-272A) and RPA32 pS (4/8) (IHC-00422) were purchased from Bethyl Laboratories (Montgomery, TX). The phospho-specific γ-H2AX antibody (JBW301) was obtained from Millipore (Billerica, MA). Mre11 (12D7), Ku70 (GTX233114) and Ku80 (GTX70485) were purchased from Genetex. Actin (C-11), BRCA1 (sc-642). RNAPII (sc-899), ATR pS 428 (sc-109912) and Rad51 (H-92) antibodies were obtained from Santa Cruz Biotech (Santa Cruz, CA). Total (2662) and pT68 Chk2 (2661) antibodies and total (2360) and pS317 (2344) Chk1 were purchased from Cell Signaling. S9.6, an antibody specific for R loops (RNA:DNA hybrids) [37], was provided by Dr. Stephen H. Leppla (NIH, Bethesda, MD). Antibodies used for ChIP: anti-BRCA1 (Gene-Tex, 6B4), anti-ATM (Novus Biologicals NB100-305), anti-γ-H2AX (Abcam, ab2893), anti-53BP1 (Novus Biologicals, NB100-305), anti-CtIP (Abcam) and anti-Gal4(DBD) (Santa Cruz Biotechnology, sc-577). Antibodies used in western blotting and SETX IP: anti-SETX (A301-105) from Bethyl Laboratories, anti-XRN2 (NBP1-68149) and anti-Rrp45 (NBP1-71702) from Novus Biologicals.

### Colony forming assays

shScr and shXRN2 cells were plated onto 60 mm tissue culture plates and allowed to grow for two days. Cells were then exposed to IR, H_2_O_2_, Aphidicolin (APH) or UV at various doses as indicated, allowed to grow for 7 days, washed with PBS and stained with crystal violet solution. Colonies with >50 normal appearing cells were counted and percent survival calculated and graphed with dose.

### Immunofluorescence

To visualize 53BP1, XRN2, and γ-H2AX, cells were plated, grown to ~70% confluency on glass coverslips and either mock- or IR-treated. Cells were then washed once with PBS, permeabilized and fixed in methanol/acetone (70/30, v/v). To visualize ATM pS 1981 and Mre11 cells were fixed in 3% paraformaldehyde/2% sucrose PBS solution for 10 min at room temperature (RT). Fixation was followed by permeabilization on ice with a 0.5% Triton X-100 buffer (0.5% Triton X-100, 20mM HEPES, pH 7.4, 50 mM NaCl, 3 mM MgCl, and 300 mM sucrose). Cells were then blocked in PBS containing 5% FBS for 30 min at room temperature. Cells were then washed three times with PBS and exposed to primary antibody for 1 h at RT as indicated. Cells were washed three times with PBS, exposed to secondary antibody for 30 min at RT, washed three times with PBS, and mounted onto glass slides. Detection of R loops using the S9.6 antibody (2 ug/ml) was performed as described [54]. Visualization was performed using a 100X oil objective lens with fluorescence on a Nikon microscope. For each experiment 100 cells were counted.

### Mammalian plasmid re-ligation assays

Were performed as described [39]. Briefly, the pEGFP-Pem1 plasmid was digested with HindIII or I-SceI for 8–12 h to generate free DNA ends. pCherry plasmids were co-transfected with linearized DNA to control for transfection efficiency. shScr and shk-h cells were transfected at ~20–25% confluency and allowed to grow for three days. Transfections were performed using Lipofectamine-2000 using the manufacturer’s instructions. Flow cytometric analyses were performed using a Beckman-Coulter Cytomic FC 500 flow cytometer.

### Metaphase spreads and chromosome aberration analyses

Exponentially growing shScr and shXrn2 cells were incubated with colcemid (1 μg/ml) for 2 h before being harvested. Harvested cells were fixed in hypotonic solution containing 75 mM KCl and fixed in methanol:acetic acid (1:1 v/v). Metaphase spreads were prepared, stained with Giemsa, and examined by light microscopy. Metaphase spreads (>50) were then scored for chromosome and chromatid aberrations as described [21].

### Nuclear extract preparation

shScr and shXRN2 cell pellets were re-suspended in Buffer A (10 mM Hepes (pH 7.9), 10 mM KCl, 0.1 mM EDTA (pH: 8.0), 0.1 mM EGTA, 1.0 mM DTT, 0.5 mM PMSF) and allowed to swell for 10 min, 4°C. NP-40 was then added to cell solutions to a final concentration of 0.5% and vortexed at low intensity for 30 sec. Isolated nuclei were then harvested by centrifugation (2,000 X g) and the nuclear pellets were re-suspended in Buffer C (20 mM Hepes (pH: 7.9), 0.4 M NaCl, 1.0 mM EDTA, 1.0 mM EGTA, 1.0 mM DTT, 0.5 mM PMSF) for 15 min at 4°C. Nuclear extracts were then isolated by centrifugation (25,000 X g, 15 min) and assessed for protein concentrations by Bradford assays.

### U87 nuclear extract preparation and SETX Immunoprecipitation

Briefly, after 2 washes with cold PBS cells were resuspended in 1 packed cell volume (PCV) of buffer A (10 mM Hepes pH 7.9, 1.5 mM MgCl2, 10 mM KCL, 0.5 mM DTT and protease inhibitors including NEM) and incubated on ice for 15 mins. The cells were passed through a 1 ml syringe 5 times and centrifuged for 20 sec. The pellet (nuclei) was resuspended in 2/3 of the PCV in Buffer C (20 mM HEPES PH 7.9, 1.5 mM MgCl2, 25% glycerol, 420 mM NaCl, 0.2 mM EDTA, 0.5 mM DTT and protease inhibitors including NEM). The extract was stirred using a mini stir bar for 30 min at 4°C. The nuclear debris were pelleted by centrifugation for 5 mins and the nuclear extract was collected in a new tube. For the IP, the glycerol was adjusted at 10% and NaCl at 150 mM. 2 μg of SETX antibody was used to IP SETX complex O/N. The IP was washed 3 times in wash buffer (10 mM Tris Hcl pH 7.4, 1 mM EDTA, 1 mM EGTA, 150 mM NaCl, 1% triton). SETX siRNA target sequence: AGCAAGAGAUGAAUUGCCA. Extracts were prepared 3 days after siRNAs transfection.

### HeLa whole cell extract preparation and gel-filtration chromatography

Were performed as described [21]. Briefly, HeLa cells were cultured in two 150 mm^2^ dishes (up to ~80% confluency) in DMEM supplemented with 5% FBS and 1 mM L-glutamine in a 5% CO_2_-95% humidified air atmosphere at 37°C. Cells were trypsinized, harvested by centrifugation and washed with ice-cold 1X PBS. Cells were re-suspended in 1 ml extraction buffer (25 mM Tris-HCl [pH: 7.7], 2 mM MgCl_2_, 100 mM NaCl, 10 mM β-glycerophosphate, 5 mM NaF, 0.5 mM Na_3_VO_4_, 10% glycerol, 0.1% NP-40, 1X protease inhibitor cocktail [Sigma], 100 units of turbonuclease [Fisher] and 1 mM DTT). Cell suspensions were incubated on ice for 5 min and passed through 1 ml syringes with 27G needles until homogeneous suspensions were obtained. Suspensions were incubated on ice for 30 min followed by 10 min at 37°C. Cell lysates were centrifuged at 14,000 rpm for 30 min at 4°C using a microfuge. Supernatants were carefully collected as whole cell lysates and used for gel-filtration chromatography. Chromatography steps were performed using AKTA Purifier 10 (GE Healthcare). For fractionation of whole cell lysates, ~3.0 mg of protein was loaded onto a 24-ml Superose 6 HR 10/30 column (GE Healthcare) pre-equilibrated with chromatography buffer (25 mM Tris-HCl [pH: 7.7], 100 mM NaCl, 5% glycerol and 1mM DTT) and run in the same buffer at a flow rate of 0.5 ml/min. Molecular weight standards (Pharmacia Biotech) were used to calibrate the column (as indicated in Fig 1C).

### DNA fiber analyses

Studies to monitor the length of DNA synthetic tracks using BrdU were performed as described [55].

### Immunoprecipitation

5 μg of specified primary antibody conjugated to Protein A/G beads. 500–1000 μg of Nuclear protein extracts were incubated with antibody:bead complex for 1 hour at 4°C. Each experiment was washed 3 times with NETN solution (20 mM Tris-HCL (pH 8.0), 0.1 M NaCl, 1 mM EDTA, 0.05% NP-40). After washes each sample was separated on 8% SDS-polyacrylamide gel.

### Chromatin immunoprecipitation (ChIP)

ChIP experiments were performed using the protocol detailed in Hatchi et al. [29]. HeLa cells were transfected with an siRNA control (sicont target sequence: UUCUCCGAACGUGUCACGU) and an siRNA targeting XRN2 (siXRN2 target sequence: GAGUACAGAUGAUCAUGUU) at 30 nM with Lipofectamine RNAiMAX (ThermoFisher) three days prior chromatin preparation. Chromatin was incubated O/N with protein G Sepharose (GE Healthcare) and the appropriate antibody: 4 μg of anti-BRCA1, 2 μg of anti-ATM, 2 μg of anti-γ-H2AX, 4 μg of anti-53BP1, 4 μg of anti-CtIP and 2 μg of anti-Gal4(DBD) used as an irrelevant antibody for control. Immunoprecipitates were then washed (with 1 ml of wash buffer for 5 min each time) twice with TSE-150 (0.1% SDS, 1% triton, 2 mM EDTA, 20 mM Tris-Hcl pH 8, 150 mM NaCl), twice with TSE-500 (0.1% SDS, 1% triton, 2 mM EDTA, 20 mM Tris-Hcl pH 8, 500 mM NaCl), once with in LiCl detergent (0.25 M LiCl, 1% NP40, 1% sodium deoxycholate, 1 mM EDTA, 10 mM Tris-Hcl pH 8) and finally once with TE. DNA was eluted from the beads with 150 μl of elution buffer (1% SDS, 100 mM NaHCO3) then supplemented with 300 mM NaCl and 10 μg/ml RNaseA and incubated for 5 hours at 65°C to reverse the crosslink. The samples were then treated with proteinase K and purified using a PCR purification kit from Qiagen. ChIP samples were analyzed by quantitative real-time PCR using Maxima SYBR green master mix from Thermo Scientific and the appropriate primers used in Hatchi et al. [29]. The results were calculated as % Input and then normalized to the negative control (Gal4(DBD) IP).

### Dot blot analysis

Genomic DNA was isolated from control and siXRN2 cells using the Dneasy Blood and Tissue Kit (Qiagen) following the manufacturer’s instructions. 50 and 100 μg of DNA was spotted directly on a nitrocellulose membrane using a Dot Blot apparatus (Bio-Rad) and UV crosslinking. Prior to blotting genomic DNA was exposed to 100 μg/ml RNase A from ThermoFisher (catalog number EN0531). RNase H treatment was performed using RNase H from New England Biolabs (catalog number M0297S) at 50 U/ml. Membrane was probed with S9.6 antibody (1 ug/ml) for 1 hour at room temp.

### Statistics

All experiments (including Western Blots and immunofluorescence images) were performed three or more times. Means and standard errors were calculated and differences between treatments were determined by confidence limit calculations using student’s t tests. p values (0.01 and 0.05) for 99% and 95% confidence limits, respectively, were considered significant and reported.

## Supporting Information

S1 Fig XRN2 undergoes transcription-dependent nuclear re-distribution in response to IR and UV. (**A-D)** Sub-cellular localization of XRN2 was monitored in fibroblast cells either (**A**) mock, (**B**) ionizing radiation (IR) (1Gy), (**C**) ultra-violet (UV) light (20 J/m2), or (**D**) UV (20 J/m2) α-amanitin (α-aman) treated cells by immunofluorescence.
(EPS)

S2 Fig XRN2 colocalizes with 53BP1 in response to UV damage. Sub-cellular localization of XRN2 and 53BP1, a DNA Damage indicator, was monitored in shSCR cells either mock (unt)- or ultra-violet (UV) light (20 J/m^2^) cells by immunofluorescence.
(EPS)

S3 Fig XRN2 relocalization after IR is independent of Kub5-Hera. XRN2 foci formation was monitored in shScr and shk-h cells either mock- or ionizing radiation (IR) (1 Gy) treated cells at times indicated by immunofluorescence.
(EPS)

S4 Fig XRN2 redistribution depends on R loops and not DSBs. Sub-cellular localization of XRN2, S9.6, and the DNA Damage indicators 53BP1 and γH2AX were monitored in either mock- or ultra-violet (UV) light at the times indicated in fibroblast cells at times indicated.
(EPS)

S5 Fig DNA Damage signaling is increased with XRN2 loss. Levels of (**A**) BRCA1, (**B**) 53BP1, (**C)** γH2AX, and (**D**) phosphorylated ATM were monitored in MCF-7 cells treated with control or XRN2 specific siRNA by immunofluorescence.
(EPS)

S6 Fig Loss of XRN2 inhibits NHEJ efficiency. (**A**) Basal levels of Rad51 foci were monitored in shXRN2 compared to shScr cells by immunofluorescence. (**B**) NHEJ efficiency was monitored in shXRN2 compared to shScr cells by NHEJ plasmid assay. (**C**) Steady-state levels of Artemis, a NHEJ core protein, was monitored by western-blot analysis in shScr, shXRN2, and shk-h cells.
(EPS)

S7 Fig Loss of XRN2 leads to increased phosphorylation of ATR and RPA32. Basal levels of phosphorylated (**A**) RPA32 and (**B)** ATR were monitored in control or XRN2 specific siRNA treated MCF-7 cells by immunofluorescence.
(EPS)

S8 Fig DSBs formed in XRN2 deficient cells are dependent on active transcription and R-loop formation. (**A, B**) Levels of 53BP1 were monitored in shXRN2 and compared to shScr cells after exposure to mock-, α-amanitin (α-aman)-, or GFP- or RNaseH-transfection treatments by IF. (**C**) Levels of R loops or 53BP1 were monitored in mock- or α-aman-treated shXRN2 cells by immunofluorescence.
(EPS)

S9 Fig XRN2 and SETX independently prevent DNA damage accumulation. (**A**) 53BP1 foci were quantitated in HeLa cells stably expressing an shRNA control (shScr) and shSETX. (**B**) Steady-state levels of phosphorylated Chk2 and γ-H2AX were monitored by western blot in the same HeLa cells analyzed in A. (**C**) Steady-state levels of SETX were measured by western blot in HeLa cells exposed to control- or XRN2-specific siRNA. (**D**) Western blot of SETX IP in U87 cell nuclear extract after transfection with siRNAs control (sicont) and targeting SETX (siSETX). Blots were probed with anti-SETX, -XRN2 and -Rrp45 antibodies. IB: Immuno-Blot, I: Input, IP: Immunoprecipitate.
(EPS)
